# Antibiotic Resistance Conferred by Class 1 Integron in Vibrio Cholerae Strains: A Meta-analysis

**DOI:** 10.24248/eahrj.v6i2.690

**Published:** 2022-11-30

**Authors:** Zavuga Zuberi, Albert Joseph Sillo

**Affiliations:** a Department of Science and Laboratory Technology, Dar es Salaam Institute of Technology, Dar es Salaam, Tanzania; b School of Life Sciences and Bioengineering, Nelson Mandela African Institution of Science and Technology, Arusha, Tanzania

## Abstract

**Background:**

Class 1 integron is the most ubiquitous platform among antibiotic resistance bacterial populations, including *Vibrio cholerae* strains. This meta-analysis aimed to determine the antibiotic resistance conferred by class 1 integron conserved segments (CS); 3′-qacEΔ1 and sul1, and 5′-int1 in *V. cholerae* strains.

**Methods:**

An intensive literature search of electronic databases for relevant studies from their starting dates up to April 2019 was conducted by two independent investigators. The electronic databases included; PubMed, Ovid Medline and Google Scholar databases. Only studies that determined antibiotic resistance conferred by class 1 integron in *V. cholerae* strains isolated from clinical and/or environmental samples using Polymerase Chain Reaction (PCR) assay were included in this study.

**Results:**

The random-effects model was selected and performed for all the studies included in this meta-analysis. Fourteen studies consisting of both qacEΔ1 and sul1, and int1 in the class 1 integron of *V. cholerae* strains were included. The proportions of class 1 integron 3′-CS and 5′-CS were 70.4 % (95%CI: 37.5–94.4) and 52 % (95% CI: 6.3–95.7) respectively.

**Conclusions:**

The proportions of class 1 integron in *V. cholerae* strains significantly contributed to the antibiotic resistances, which are comparable to other gram-negative bacteria clinical isolates. Moreover, the 3′-CS qacEΔ1 and sul1 are highly involved in the antibiotic resistance in comparison to 5′-CS int1. Generally, the study findings provide a general view on antibiotic resistance conferred by class 1 integron in *Vibrio cholerae* strains.

## BACKGROUND

Cholera is a disease with the most rapidly devastating effects, accounting for morbidity, mortality, and antibiotic drug resistance in people's life.^1^*Vibrio cholerae* are environmental organisms that can acquire antibiotic-resistant genes through intimate contact with fundamentally resistant environmental bacteria using mobile genetic elements that share resistant traits with other enteric pathogens.^2,3^ This results in antibiotic resistance of *V. cholerae* strains to drugs, thus leading to the high burden of cholera disease in different parts of the world.^4,5^

**FIGURE 1:**
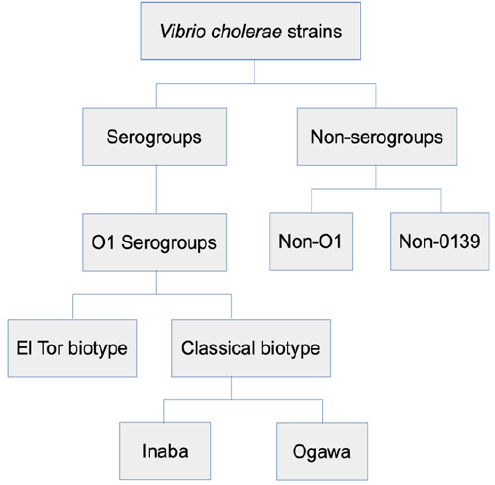
Classification of Vibrio Cholerae Strains

Antibiotic-resistant genes are carried in class 1 integron elements capable of moving resistant genes and integrating them into chromosomes of the bacteria by site-specific recombination.^9^ Class 1 integron has two conserved segments (CS); 3′-CS and 5′-CS with variable region possessing antibiotic resistance gene cassettes.^10^ The 3′-CS segment contains the *qac*EΔ1 and *sul*1 genes possessing 800 bp amplicon size while 5′-CS, an integrase (*int*I), and its attachment site (*att*I) are located together with gene promoter with about 900 bp amplicon size.^3^ The molecular variation in amplicon size for 5′-CS *int*I can be due to several factors, including primer degradation, primer slippag, polymerase dissociation, and mis-priming due to the secondary structure accounting for the differences.^11,12^ About 200 O-serogroups of *Vibrio cholerae* strains exist but only serogroup O1 is associated with antibiotic resistance. The serogroup O1 is classified into classical and E1 Tor biotypes.^5^ The classical biotype is further classified into O1-Inaba, and O1-Ogawa biotypes (Figure 1). The O1-Inaba, O1-Ogawa, and El Tor biotypes share genomic properties between their biotype strains. The most apparent difference between O1 serogroup and non-serogroups (non-O1 and non-O139) relies on the possession of a capsule.^6,7^ Serogroups other than O1 and O139 are generally named *V. cholerae* non-O1, non-O139, or non-agglutinating Vibrios (NAGs).^8^

Different molecular characterization techniques have been reported for determining the *V. cholerae* serogroup 1 from clinical and environmental samples. These include multi-locus enzyme electrophoresis, ribotyping, polymerase chain reaction (PCR) assays, and pulsed-field gel electrophoresis.^6^ However, other molecular techniques such as simplex and multiplex PCRs can be used for non-O1, and non-O139 *V. cholerae*.^8^

Despite the diversity of molecular techniques, all studies included in this meta-analysis used PCR assay in studying antibiotic resistance in *V. cholerae* strains as it can detect CS amplicons of smaller-size ranging between 800 and 900 bp.

Resistant serogroup O1 of *V. cholerae* has been disseminated globally, which threatens the effective treatment and control of cholera mostly in low- and middle-income countries.^13^ However, there are limited data available about the nature and extent of antibiotic resistance caused by the serogroup O1 strains.^14^ Although most of the studies about the intervention of Cholera and its antibiotic resistance were done in other parts of the world like India, Iran, and China. Africa is also known to be affected by this pandemic. Cholera was imported to Africa through West Africa and then spread to East, Central, and later South Africa in the 1970s during the seventh pandemic.^28^ Most prominently El Tor and classical biotypes were later identified in clinical and environmental samples in different parts of Africa. This necessitates the need to conduct in-depth studies of antibiotic resistance for these biotypes as one of the management strategies for Cholera intervention in Africa. Moreover, Mohammed et al.^28^ reported in their systematic review done in sub-Saharan African countries that among the antibiotics resisted by *V. Cholerae* the most reported includes in their order of Trimethoprim, Sulphamethoxazole, Ampicillin, Chloramphenicol, and Streptomycin. However, very little information is known concerning the magnitude of antibiotic resistance conferred by class 1 integron in *Vibrio cholerae* strains. Therefore, this study determined the antibiotic resistance conferred by class 1 integron conserved segments (CS); 3′-*qacEΔ*1 and *sul*1, and 5′-*int*1 in *V. cholerae* strains.

## METHODS

### Overview of the Modus Operandi for the Study

This meta-analysis was performed following the Preferred Reporting Items for Systematic Reviews and Meta-Analyses (PRISMA) guidelines.^30^A comprehensive literature search of studies that meet the inclusion criteria was conducted in the 3 electronic databases.

### Search Strategy

The intensive literature searches of the relevant studies from their starting dates to April 2019 were conducted in PubMed, Ovid Medline, and Google Scholar databases. The terms ‘class 1 integron' ‘antibiotic resistance', and ‘*Vibrio cholerae*' were used in the searching. These searches were supplemented by scanning citations for the relevant studies. All identified study abstracts were independently reviewed for their eligibility by two investigators.

### Inclusion and Exclusion Criteria

Studies which were included in the meta-analysis met the following criteria: (1) conducted on either clinical or environmental samples of *V. cholerae* strains; (2) used PCR assay to identify antibiotic resistance conferred by class 1 integron in *V. cholerae* strains studies; (3) full-text articles accessed; and (4) article written in English language. Studies which used phenotypic methods instead of PCR assay, reported review, systematic reviews or meta-analyses of other studies, congress abstracts and those written in languages other than English were excluded.

### Selection Procedure

The titles and abstracts of all searched records were reviewed to identify the full-text articles for eligibility and determine their relevance for inclusion in the meta-analysis.

### Data extraction

Reviewers used a standardized data extraction form to extract data from studies which met the inclusion criteria.^29^ Where there was disagreement, a discussion between the two reviewers was conducted so as to reach a consensus. The extracted information included author's name, publication year, country, the total number of *V. cholerae* strains studied, type of *V. cholerae* strains, study period, number of strains with antibiotic resistance, and the relative frequency of *V. cholerae* strains.

### Minimizing Biases

To minimize bias, two authors reviewed the articles independently and the retrieved records were double-checked. Publication biases are presented in funnel plots in Figure 4.

### Statistical Analysis

All statistical analyses were performed using MedCalc Statistical Software (18.11.6; MedCalc Software bvba, Ostend, Belgium). The weighted random-effects model for each study sample size was considered statistically significant at *p*<0.05. In addition, the pooled proportions and 95% confidence intervals (CIs) for positive *V. cholerae* strains per class 1 integron CS were computed using proportions presented in the full-text report of each study. Heterogeneities among studies were evaluated using the I ^2^ statistic with 95% CI. For publication bias, funnel plots were derived for each class 1 integron CS (Figure 4).

## RESULTS

### General Characteristics of Studies Involved in the Analysis

**FIGURE 2:**
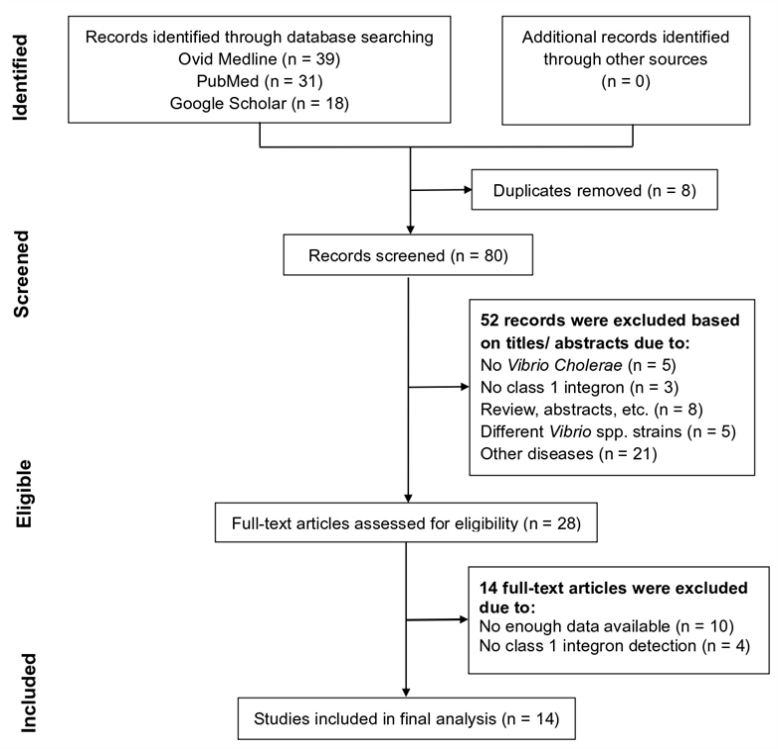
Study Flow for the Literature Inclusion and Exclusion

Using three electronic databases and manual searches yielded 88 references. However, 8 references were excluded as they were duplicate publications. Based on titles and/or abstracts, we excluded 52 references and reviewed 28 references for full-text articles. After the application of the study inclusion criteria, 14 studies of antibiotic resistance in *V. cholerae* in the years between 1996 and 2016 were included in the meta-analysis (Figure 2). The pooled studies that were included in our meta-analysis involved O1-Inaba, O1-Ogawa, El Tor, non-O1, and non-O139 of *V. cholerae* strains and were studied to determine the antibiotic resistance conferred by class 1 integron.

### Number of *V. Cholerae* Strains with Antibiotic Resistance

Moreover, 10 articles studied *qacE*Δ1 and *sul*1^9, 10, 14–21^, 6 articles studied *int*1^4, 10, 16, 22–24^, and 2 articles studied both *qacE*Δ1 and *sul*1; and gene cassette *int*1^10, 16^ in the class 1 integron of *V. cholerae* strains. The amplicon sizes reported in the studies were 800 bp for *qacE*Δ1 and *sul*1, while *int*1 varied between 900-1800 bp (Table 1). All studies involved clinical *V. cholerae* strains; except one study that included only environmental *V. cholerae* strains.^22^

**TABLE 1:** Studies Included in Meta-Analysis

Study [ref]	Publication year	Study period	Country	*V. cholerae* strains studied	Number of *V. cholerae* strains	Number of antibiotic resistant strains	Class 1 integron probes	Amplicon size (bp)
Adabi et al. [9]	2009	2004-2006	Iran	O1-Inaba, O1-Ogawa, non-O1, non-O139	60	1	qacEΔl-F and sull-R	800
Bakhshi et al. [22]	*2009	2006	Iran	Non-O1, non-O139	37	2	intl-F, intl-R	ns
Bakhshi et al. [23]	2014	3 years duration	Iran	Ns	24	1	intl-F, intl-R	900
Dalsgaard et al. [16]	1999	1979-1990	Vietnam	O1-0gawa^a^	34	20	qacEΔl-F, sull-B	800
				O1-Inaba^b^	20	20	intl-F, intl-B	1,000
Dalsgaard et al. [15]	2000	1982-1995	Thailand	O-Serotype	176	44	qacEΔl-F, sull-B	800
Dalsgaard et al. [16]	2000	1996-1997	Guinea-Bissau	O1-Serotype	91	46	qacEΔl-F, sull-B	800
					46	46	intl-F, intl-B	1,800
Dalsgaard et al. [14]	2001	1997-1998	Mozambique & South Africa	O1-Ogawa	20	19	qacEΔl-F, sull-B	800
Goel et al. [17]	2010	2004	India	O1-Ogawa	44	44	qacEΔl-F, sull-B	800
Goel et al. [18]	2010	2004-2007	India	O1-Ogawa	114	114	qacEΔl-F, sull-B	800
Jain et al. [20]	2008	2007	India	O1-Bl Tor	32	32	qacEΔl-F, sull-B	800
Jain et al. [19]	2011	2010	India	O1-Ogawa	41	41	qacEΔl-F, sull-B	800
Mala et al. [4]**	2016	2004-2012	Thailand	01, non-O1, non-O139	92	1	intl-F and intl-R	923
Mwansa et al. [24]	2007	1990-2004	Zambia	O1-Bl Tor	69	22/23^c^ 3/4^d^	intl-F and intl-R	923
Shi et al. [21]	2006	1992-2000	India	01, 0139, non-O1, non-O139	133	14	qacEΔl-F, sull-B	800

* study involved V. cholerae strains from environmental samples

** study involved 67 V. cholerae strains from clinical samples and 25 V. cholerae strains from environmental samples

a astrains isolated between 1979-1981

b strains isolated between 1982-1999

c and d strains isolated in the year 1996 and 1997 respectively

AR: Antibiotic resistance

ns not stated

### Heterogeneity and Publication Bias

**FIGURE 3:**
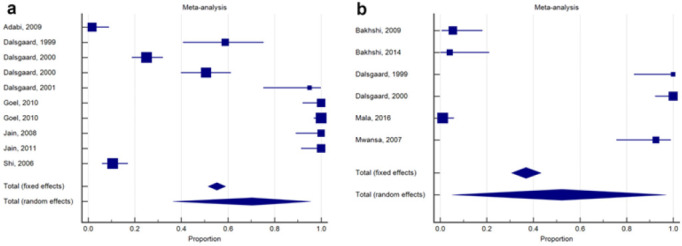
Forest plot for the antibiotic resistance conferred by (a) class 1 integron qacEΔ1 and sul1 3′-CS (b) class 1 integron int1 5′-CS in Vibrio cholerae strains Each square is proportional to the percentage weight of each study in the meta-analysis. The diamond represents the overall summary estimate, with the confidence interval given by its width.

**FIGURE 4:**
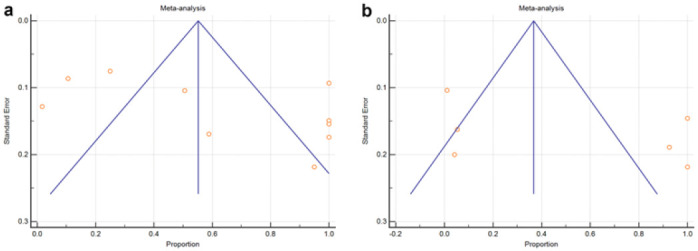
The funnel plot for the antibiotic resistance was conferred by (a) class 1 integron qacEΔ1 and sul1 3′-CS (b) class 1 integron int1 5′-CS in Vibrio cholerae The vertical line in the middle represents the estimate of summary effect size and the two stands sidelined show the spread of the 95% CIs. Each point represents a separate study.

On average proportions of class 1 integron 3′-CS and 5′-CS were (70.4 %; 95% CI: 37.5–94.4), and (52 %; 95% CI: 6.3–95.7), respectively (Figure 3a, Table 2). Heterogeneities between studies were high for the class 1 integron *qacE*Δ1 and *sul*1 (I^2^: 98.8 %; 95% CI: 98.5–99.1, *P*<0.0001); while for *int*1 (I^2^: 98.7 %; 95% CI: 98.1–99.1, *P*<0.0001) (Figure 3b, Table 2). Concerning publication bias, the funnel plots of *qacE*Δ1 and *sul*1 3′-CS, and *int*1 5′-CS of class 1 integron in *V. cholerae* strains indicate the symmetrical distribution in the absence of bias (Figure 4).

**TABLE 2:** Meta-analysis of Antibiotic Resistance and Heterogeneity Test For Class 1 Integron Conserved Segments

Study [ref.]	Number of *V. cholerae* strains	Number of antibiotic resistant strains	Percent antibiotic resistance	95% CI	Weight (%)	I^2 (95% CI)
**Meta-analysis: % antibiotic resistance by qacEΔ1 and sul1 3′-CS**						
Adabi, 2009 [9]	60	1	1.7	0.04-8.9	10.0	98.8% (98.5-99.1)
Dalsgaard, 1999 [16]	34	20	58.8	40.7-75.3	9.9	
Dalsgaard, 2000 [15]	176	44	25.0	18.8-32.1	10.1	
Dalsgaard, 2000 [10]	91	46	50.5	39.9-61.2	10.1	
Dalsgaard, 2001 [14]	20	19	95.0	75.1-99.9	9.8	
Goel, 2010 [17]	44	44	100.0	91.9-100.0	9.9	
Goel, 2010 [18]	114	114	100.0	96.8-100.0	10.1	
Jain, 2008 [20]	32	32	100.0	89.1-100.0	9.9	
Jain, 2011 [19]	41	41	100.0	91.4-100.0	9.9	
Shi, 2006 [21]	133	14	10.5	5.9-17.0	10.1	
Total (random effects)	745	375	70.4	37.5-94.4	100.0	
**Meta-analysis: % antibiotic resistance by inti 5′-CS**						
Bakhshi, 2009 [22]	37	2	5.4	0.7-18.2 16.70	98.7%	(98.1-99.1%)
Bakhshi, 2014 [23]	24	1	4.2	0.1-21.1 16.58	16.52	
Dalsgaard, 1999 [16]	20	20	100.0	83.1-100.0	16.52	
Dalsgaard, 2000 [10]	46	46	100.0	92.3-100.0	16.74	
Mala, 2016 [4]	92	1	1.1	0.03-5.9 16.84		
Mwansa, 2007 [24]	27	25	92.6	75.7-99.1	16.62	
Total (random effects)	246	95	52.2	6.3-95.7 100.00		

## DISCUSSION

Cholera outbreaks are seasonally ongoing in some developing countries. For decades, antibiotic resistance patterns have not been well studied and elucidated.^25^ This consequently affects the treatments for disease, leading to high mortality rates during outbreaks. Our study aimed at demonstrating the current perspectives of antibiotic resistance for class 1 integron in *Vibrio cholerae*. Most studies were conducted in India^5^, Iran^3^, Thailand^2^, Vietnam^1^, Guinea-Bissau^1^, Mozambique & South Africa^1^ and Zambia^1^.

Published information on the meta-analysis of antibiotic resistance conferred by class 1 integron in *Vibrio cholerae* strains are limited. Only one meta-analysis was published generally on gram-negative bacteria clinical isolates.^26^ It included 29 studies which were conducted in Iran, and evaluated the prevalence of integron classes and different gram-negative bacterial strains. Our meta-analysis determined the effects of antibiotic resistance conferred by class 1 integron CS 3′-*qacEΔ*1 and *sul*1, and 5′-*int*1 in *V. cholerae* strains among countries that had cholera outbreaks. There was a significant presence of integrons in clinical isolates with a pooled prevalence of (79%; 95% CI 73.6–83.7) of class 1 integrons in multi-drug resistance (MDR) isolates which is comparable to the pooled proportion of 70.4% in class 1 integron 3′-CS reported herein.^26^ In addition, this showed independent effects of antibiotic resistance of conserved segments in class 1 integron in *V. cholerae* strains.

Moreover, of all the pooled studies, 71.4% antibiotic resistance was highly contributed by the conserved segment 3′-CS *qacE*Δ1 and *sul*1 as compared to the 42.9 % antibiotic resistance outcomes in the conserved segment *int*1 5′-CS (Table 2). In addition, the majority of the O1 serogroups identified by this study are supported by another meta-analysis study that reported 80.0% of the predominating cholera toxigenic *V. cholerae* isolates of the serogroup O1 were the El Tor biotype with Ogawa and Inaba serotypes.^28^ There are several factors that might have contributed to the antibiotic resistance caused by class 1 integron in *V. cholerae* across different countries. Some of the potential factors may include the exportation of drugs via efflux pumps, chromosomal mutations or the exchange of conjugative plasmids, conjugative transposons, integrons, or self-transmissible chromosomally integrating SXT elements.^27^

### Limitations of the Study

Some possible limitations should be considered for this meta-analysis. First, the limited literature search to only studies published in the English language. This may be associated with some systematic bias in our meta-analysis that might have a significant impact on the study findings. Second, heterogeneities exist among studies included in this meta-analysis. Although the random-effects model allows the presence of heterogeneity, there may still be disagreement regarding the pooled estimates proportions in the presence of heterogeneity among studies. Finally, the limited number of studies that met eligibility criteria could have possibly affected the statistical analyses in detecting funnel plot symmetry in reporting biases.

## CONCLUSION

This meta-analysis study has provided a general view on antibiotic resistance conferred by class 1 integron of *Vibrio cholerae*. Our study highlights the proportions of antibiotic resistance determined by conserved regions (3′-CS and 5′-CS) that can be used for monitoring and developing control strategies. However, a very limited number of studies have focused on antibiotic resistance against *Vibrio cholerae* strains. Therefore, more research on the detection of class 1 integron as a remarkable genetic platform is highly recommended. There is also a need for developing new control strategies and involvement of experts in the relevant field in the management of antibiotic resistance among *V. cholerae* strains.
